# Progress towards elimination of measles in Kenya, 2003-2016

**DOI:** 10.11604/pamj.2018.31.65.16309

**Published:** 2018-09-28

**Authors:** Ngina Kisangau, Kibet Sergon, Yusuf Ibrahim, Florence Yonga, Daniel Langat, Rosemary Nzunza, Peter Borus, Tura Galgalo, Sara A Lowther

**Affiliations:** 1 Field Epidemiology and Laboratory Training Program, Kenya; 2 World Health Organization, Immunization and Vaccines, Kenya; 3 Ministry of Health, Division of Disease Surveillance and Epidemic Response, Kenya; 4 Kenya Medical Research Institute, Centre for Virus Research, Kenya; 5 World Health Organization, Measles and Polio Laboratory, Kenya; 6 United States Centers for Disease Control and Prevention, Kenya; 7 Division of Global Health Protection, Center for Global Health, Centers for Disease Control and Prevention, Atlanta, USA

**Keywords:** Measles, surveillance, elimination, Kenya

## Abstract

**Introduction:**

Measles is targeted for elimination in the World Health Organization African Region by the year 2020. In 2011, Kenya was off track in attaining the 2012 pre-elimination goal. We describe the epidemiology of measles in Kenya and assess progress made towards elimination.

**Methods:**

We reviewed national case-based measles surveillance and immunization data from January 2003 to December 2016. A case was confirmed if serum was positive for anti-measles IgM antibody, was epidemiologically linked to a laboratory-confirmed case or clinically compatible. Data on case-patient demographics, vaccination status, and clinical outcome and measles containing vaccine (MCV) coverage were analyzed. We calculated measles surveillance indicators and incidence, using population estimates for the respective years.

**Results:**

The coverage of first dose MCV (MCV1) increased from 65% to 86% from 2003-2012, then declined to 75% in 2016. Coverage of second dose MCV (MCV2) remained < 50% since introduction in 2013. During 2003-2016, there were 26,188 suspected measles cases were reported, with 9043(35%) confirmed cases, and 165 deaths (case fatality rate, 1.8%). The non-measles febrile rash illness rate was consistently > 2/100,000 population, and “80% of the sub-national level investigated a case in 11 of the 14 years. National incidence ranged from 4 to 62/million in 2003-2006 and decreased to 3/million in 2016. The age specific incidence ranged from 1 to 364/million population and was highest among children aged < 1 year.

**Conclusion:**

Kenya has made progress towards measles elimination. However, this progress remains at risk and the recent declines in MCV1 coverage and the low uptake in MCV2 could reverse these gains.

## Introduction

Elimination of measles is defined as the absence of endemic measles cases in a country or region for ≥12 months while under adequate surveillance [1-3]. Measles has been successfully eliminated in the Americas [4]. All other World Health Organization (WHO) regions have set elimination goals for measles. In 2011, the WHO African Region (AFR) adopted a measles elimination goal to be reached by the end of 2020 [5]. Activities for measles control and elimination include routine immunization (RI), supplemental immunization activities (SIAs), case management, outbreak response, and surveillance [5]. RI builds protection against measles virus by providing two doses of a measles-containing vaccine (MCV) to infants aged 9-18 months [6]. SIAs are immunization campaigns targeting children in a defined age group in a specified geographical area. SIAs provide a second opportunity for measles immunization in children not reached by RI or those not seroconverting after routine immunization [7, 8]. “Catch-up” SIAs are usually the initial campaigns conducted, targeting children aged 9 months through 14 years, with the aim to achieve high population immunity quickly and interrupt measles transmission, while “follow-up” SIAs are conducted at regular intervals, usually 3-4 years, among children aged <5 years, to reduce accumulation of susceptible children [9]. The WHO Africa Regional Office (AFRO) has established measles elimination indicators, including: 1) measles incidence <1 case per million population at national level; 2) ≥95% MCV1 coverage in RI at national and at sub-national levels; 3) 95% coverage in measles SIAs and outbreak response immunization activities (ORI); 4) ≥80% of districts (sub-national levels) investigating one or more suspected measles cases within a year; and 5) reported non-measles febrile rash illness rate of ≥2 per 100,000 population at national level [5]. In Kenya, the Expanded Program on Immunization (EPI) was started in 1980 with the first dose of measles-containing vaccine (MCV) or MCV1 given at 9 months of age. Accelerated control for measles was started in 2002 when the first catch-up SIA that targeted children aged 9 months to 14 years was conducted, followed by periodic SIAs and the establishment of a case-based surveillance for measles with laboratory confirmation. In addition, the Reach Every District approach has been implemented in the country since 2003 to improve RI coverage. With the promulgation of the new constitution in 2010, Kenya adopted a decentralized government and replaced the eight provinces and existing districts with 47 semi-autonomous counties. In 2013, health services were fully decentralized to the counties to run the devolved functions, following the election of governors. Measles case-based surveillance therefore shifted from being district-reported to county-reported. A second dose of MCV (MCV2) was introduced in 2013 and is recommended for children aged 18 months [10]. Kenya developed a national measles elimination strategic plan 2012-2020 as a road map for eliminating measles transmission. This analysis aims to describe measles immunization performance, the surveillance performance against WHO standards, the epidemiology of measles cases in Kenya and to track the progress towards elimination of measles.

## Methods

We conducted a review of RI data, SIAs data and the Kenya national measles case-based surveillance data for the period between January 2003 and December 2016. Data on RI were obtained from the administrative data maintained by the Kenya Ministry of Health National Vaccine and Immunization Program (NVIP). Data on measles SIAs were obtained from the post-campaign reports. Surveillance data were obtained from a Microsoft Access database maintained at the disease surveillance and response unit. Population estimates were projected from the national census data from the Kenya National Bureau of Statistics (KNBS). In Kenya, once a clinician suspects measles, he/she notifies the sub-county disease surveillance coordinator (SCDSC) who completes a case-based form in duplicate and collects a blood specimen for laboratory testing. Both specimen and completed form are sent to the Kenya Medical Research Institute (KEMRI) measles laboratory, the WHO-accredited national laboratory, and case details are entered into a central database. A measles outbreak is suspected when five suspected cases are reported from a health facility or sub-county within a month. An outbreak is confirmed when three suspect cases have been laboratory confirmed. In measles case-based surveillance, a suspected measles case is defined as occurrence of fever and a maculopapular rash with any one of cough, coryza, or conjunctivitis or any illness in a person that a clinician suspects to be measles. Confirmed measles cases can be classified as laboratory-confirmed, epidemiologically linked, or clinically compatible. A suspected measles suspect case is laboratory-confirmed when measles-specific IgM antibody is detected in serum by enzyme linked immunosorbent assay (ELISA) in the absence of measles vaccination within 30 days of before specimen collection. A suspect measles case is epidemiologically linked to a confirmed case when the patient has had contact with or lives in the same locality as a person with laboratory-confirmed measles infection. A suspect case is clinically-compatible when it meets the clinical case definition but a laboratory test or an epidemiological link are is lacking [11, 12]. For this analysis, we included all laboratory-confirmed, epidemiologically linked, and clinically compatible measles cases as confirmed measles cases.

Microsoft Excel was used for managing and cleaning the database for analysis. Variables analyzed included age, sex, immunization status, date of rash onset, date seen at facility, date of notification, specimen quality, duration of processing, laboratory results, county of origin, and outcome. Analysis was done using Epi-Info 7, and maps were generated with Arc GIS Version 10.0. Surveillance indicators were calculated in accordance with WHO standard guidelines. They included the following: 1) national-level non-measles febrile rash illness (NMFRI) rate (target of ≥2 NMFRI cases per 100,000 population); 2) proportion of sub-counties reporting one or more rash illness case per year (target ≥80% sub-county units); 3) timeliness of specimen arrival to laboratory (defined as 80% arriving within 3 days of collection); and 4) timeliness in feedback of results (defined as at least 80% of results sent back within 7 days of arrival of specimen to laboratory). Measles incidence was calculated as cases per million populations, using Kenya National Bureau of Statistics population estimates for the respective year. Case fatality rate (CFR) was calculated for measles deaths with confirmed cases as denominator and confirmed case deaths as numerator. This study was determined to be routine analysis of surveillance data by the disease surveillance and response unit and did not require a full ethics review. Permission to conduct the analysis was obtained from the Head, Disease Surveillance and Response Unit, Ministry of Health, Kenya. This analysis of surveillance data was classified as non-research by the Centers for Disease Control and Prevention (CDC) Center for Global Health.

## Results

**Figure 1:**
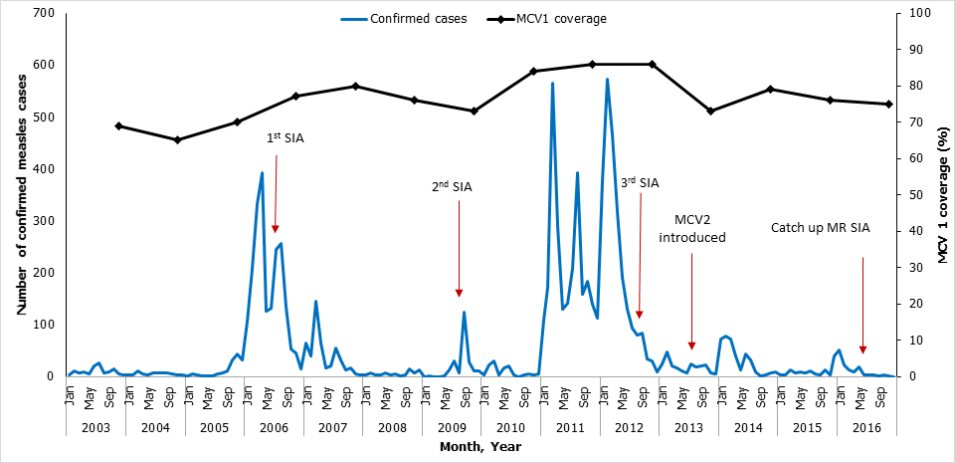
Distribution of confirmed measles cases, measles containing vaccine (MCV) coverage and key measles control events by year-Kenya, 2003-2016

**Routine immunization coverage:** Administrative MCV1 coverage increased from 69% in 2003 to 86% in 2012, subsequently declined to 73% in 2013, and then slightly improved to 75% in 2016 (Figure 1). Since introduction in mid-2013, MCV2 uptake has increased slowly, from 15% in 2014 to 26% in 2015 and 32% in 2016.

**Supplemental immunization activities:** The first nationwide catch-up campaign using monovalent measles vaccine was conducted in 2002; it targeted 14 million children aged 9 months to 14 years and achieved a coverage of 94%. Subsequently, four SIAs were conducted in Kenya during 2003-2016 (Figure 1). Monovalent measles vaccine was used for the first three, which were follow-up campaigns. The first SIA was done in 2006; it targeted 4.7 million children aged 9-59 months, was conducted in 2 phases, and achieved 111% national reported administrative coverage. The second SIA was conducted in 2009, targeting 6.7 million children aged 9-59 months, and achieved an administrative coverage of 82%. The third SIA, conducted in 2012, targeted 6 million children aged 9-59 months and achieved 100% administrative coverage and 92% coverage by survey. In 2016, the country conducted a catch-up campaign with measles-rubella (MR) vaccine, targeting 19 million children aged 9 months to 14 years, and achieved a reported 96% coverage. The last SIA was followed by national introduction of MR vaccine into routine immunization schedule in late 2016.

**Case-based surveillance indicators:** During the study period, the national non-measles febrile rash illness (NMFRI) reporting rate was ≥2 cases per 100,000 persons all 14 years, and the proportion of counties investigating >one suspect case per year was ≥80% in all but 3 years (Table 1). A high proportion of suspected measles cases were investigated with collection of an adequate serum specimen for laboratory testing. However, there was delay in transportation of collected samples to the laboratory within the targeted period (≤3 days), and there was delay in reporting of laboratory results to the sub-national levels (Table 1).

**Table 1:** Performance of measles surveillance indicators by year -Kenya 2003-2016

Selected Measles Surveillance Indicators:	Target	Year of surveillance **[** **1** **]**															
		2003	2004	2005	2006	2007	2008	2009	2010	2011	2012	2013	2014	2015	2016	Mean	Years target met (N=14)
Non measles febrile rash rate per hundred thousand population	>2	6	12	10	10	9	9	8	8	9	8	7	8	7	2	8	14
Proportion of counties investigating ≥1 case of measles per year	≥80%	88	88	90	96	91	80	81	50	60	84	62	85	86	94	81	11
Proportion of suspected cases lab investigated	≥80%	99	99	99	98	93	87	99	99	100	100	100	100	100	100	98	14
Proportion of adequate specimen	≥80%	96	98	97	96	92	85	98	99	100	100	99	99	100	98	97	14
Proportion of specimen arriving at lab within 3 days	≥80%	61	53	59	71	65	64	69	67	63	62	59	63	61	60	63	0
Proportion of specimen with results within 7 days	≥80%	40	64	68	66	86	83	87	93	79	95	94	84	72	93	79	8
Number of indicators meeting target	6	4	4	4	4	5	5	5	5	3	5	4	5	4	5	4	

[1] Year refers to calendar year from January to December

**Figure 2:**
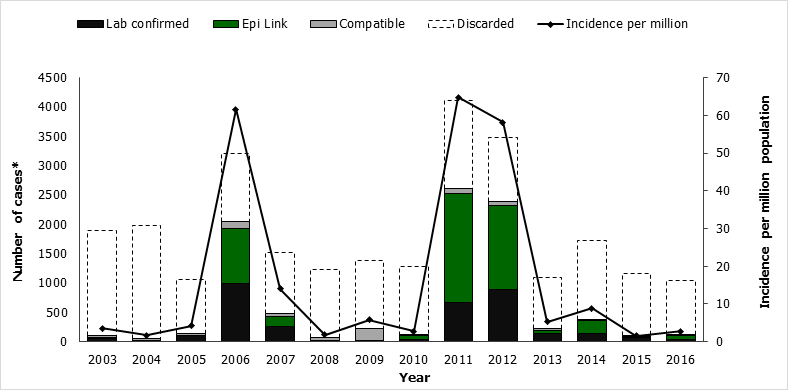
Classification of reported cases and incidence of confirmed measles cases-Kenya, 2003-2016

**Epidemiology of measles cases:** A total of 26,188 suspected measles cases were notified through the Kenyan national measles case-based surveillance system from January 2003 to December 2016. Of these, 9,043 (35%) cases were confirmed measles cases, including 3,423 (38%) laboratory confirmed, 4,856(54%) epidemiologically linked, and 764 (8%) clinically compatible cases. The proportion of discarded cases remained constant over the years including the years with outbreaks (Figure 2). The overall incidence for the 14-year period was >5 cases/million; annual incidence ranged from 2-65 cases/million persons (Table 2, Figure 2). There were no significant differences in measles incidence by gender. The highest incidence rates occurred among infants aged <1 year (overall: 76; range: 1-349) and children aged 1-4 years (overall: 55; range: 0.1-364), and among those in urban residences (overall: 118; range: 3-886). Among confirmed cases, coverage with ≥one dose of MCV ranged from 20%-77%; overall, only 3,069 (34%) of cases had received one or more doses (Table 1). Among cases, 3,316 (36%) were hospitalized, and there were 165 deaths reported during the study period (Case Fatality Rate 1.8%).

**Table 2:** Demographic and epidemiologic characteristic of confirmed measles cases by year-Kenya, 2003-2016

Characteristic	Year														
	2003 No.(IR^1^)	2004 No.(IR)	2005 No.(IR)	2006No.(IR)	2007 No.(IR)	2008 No.(IR)	2009 No.(IR)	2010 No.(IR)	2011 No.(IR)	2012 No.(IR)	2013 No.(IR)	2014 No.(IR)	2015 No.(IR)	2016 No.(IR)	Total(IR)
**Confirmed measles^2^**	113(4)	56(2)	139(4)	2042(62)	474(14)	65(2)	222(6)	110(3)	2608(65)	2380(58)	223(5)	375(9)	111(2)	125(3)	9043
**Sex**															
Male	50(3)	28(2)	56(3)	1030(62)	240(14)	35(2)	111(6)	79(4)	1349(66)	1237(59)	108(5)	214(10)	58(1)	73(3)	4668(17)
Female	63(4)	28(2)	83(5)	972(59)	229(14)	30(2)	110(6)	29(1)	1252(62)	1142(52)	115(5)	161(7)	53(1)	48(2)	4315(16)
Missing	0	0	0	40	5	0	1	2	7	1	0	0	0	4	60(1)
**Age group**															
< 1 year	8(7)	1(1)	19(15)	424(337)	79(61)	14(11)	14(11)	4(3)	227(177)	460(349)	68(50)	56(40)	3(2)	9(6)	1386(76)
1–4 years	45(12)	25(7)	53(14)	631(159)	101(25)	27(7)	77(16)	18(4)	782(157)	1854(364)	0	1(0.1)	14(3)	36(6)	3664(55)
5–14 years	47(5)	22(2)	37(4)	443(48)	107(11)	22(2)	103(10)	66(6)	892(80)	1	0	2(0.1)	6(0.1)	33(3)	1781(12)
15+ years	13(0.7)	6(0.3)	9(0.5)	474(26)	171(9)	2(0.1)	66(0.8)	15(0.7)	604(26)	0	0	244(10)	87(3)	35(1)	1677(6)
Missing	0	2	21	70	16	0	0	7	103	65	155	72	1	12	535
**Vaccination status^3^**															
0 dose	6(5)	5(9)	63(45)	475(23)	183(39)	11(17)	16(7)	54(49)	950(36)	871(37)	81(36)	120(32)	30(27)	28(22)	2893(32)
≥ 1 dose	77(68)	43(77)	42(30)	408(20)	142(30)	33(51)	159(72)	35(32)	879(34)	775(33)	98(44)	255(68)	80(72)	43(34)	3069(34)
Unknown	30(27)	8(14)	34(25)	1159(57)	149(31)	21(32)	47(21)	21(19)	779(30)	734(31)	44(20)	0	1(1)	54(43)	3081(34)
**Residence**															
Rural	59(3)	22(1)	0	167(7)	181(8)	5(0.1)	39(1)	0	1454(52)	2084(73)	193(7)	259(9)	97(2)	100(3)	4660(9)
Urban	24(24)	31(30)	3(3)	372(351)	20(18)	2(2)	3(2)	0	1153(886)	296(222)	26(19)	116(83)	10(7)	5(3)	2061(118)
Missing	30(27)	3(6)	136(98)	1503(74)	273(58)	58(89)	180(81)	110(100)	1	0	4(1)	0	4(4)	20(16)	2322(26)

^[1]^ IR is Incidence Rate, calculated by dividing the number of confirmed cases by population estimate for the year and multiplied by 1,000,000

2 Confirmed cases include laboratory-confirmed, epidemiologically confirmed and clinical-compatible case

3 Vaccination status data on parentheses is percentage

**Figure 3:**
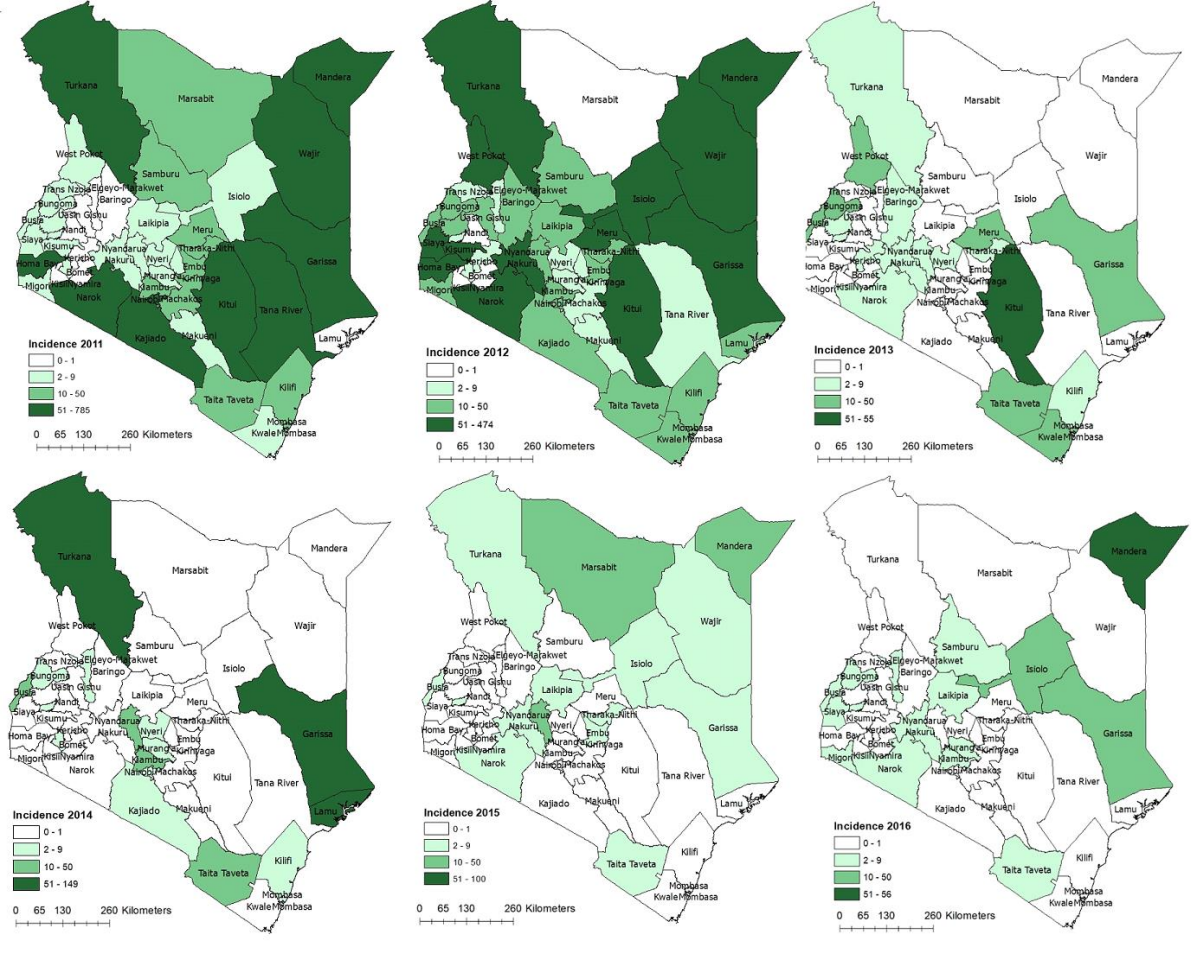
Spatial trend in incidence of measles by county-Kenya, 2011-2016

**Trends in confirmed measles cases:** The epidemiology of measles in Kenya over the past 14 years has been characterized by periodic outbreaks and improved control after SIAs (Table 2, Figure 1, Figure 2). Following the catch-up campaign of 2002, the incidence of measles cases was <5/million population during 2003-05. In 2006, an outbreak occurred with 2042 confirmed cases (incidence of 62/million). Infants and children aged <5 years had the highest incidence, but 45% of the confirmed cases occurred in children aged 5-14 years and adults >15 years old. After the 2006 follow-up SIA, measles incidence ranged from 2-6/million population during 2008-2010. Another large outbreak occurred in 2011-2012, with nearly 5,000 cases, 67% of which occurred in infants and children <5 years old. It was preceded by declining MCV1 coverage and suboptimal coverage during the 2009 follow-up SIA. All 47 counties reported cases during these two nationwide outbreaks, and they accounted for 7,003 (78%) of all confirmed cases during the 14-year period. Smaller outbreaks were detected in 2009 (10 counties affected) and during 2013-2015 (21 counties affected), after the 2012 follow-up SIA. Of these 21 counties, 9 reported an outbreak in 2013, 7 in 2014, 4 in 2015 and 1 in 2016. Measles incidence was highest in 2011 at 65/million population and declined to 1.6/million population in 2015. The proportion of counties with an incidence exceeding the elimination threshold (1/million) decreased between 2011 and 2016 (Figure 3).

## Discussion

This analysis of measles immunization data and measles case-based surveillance indicates that Kenya has made progress toward measles elimination but the progress has been uneven and the country is still at risk of not attaining measles elimination goal. During this period, Kenya maintained case-based surveillance despite disruptions such as the decentralization of government and the devolution of health functions to county governments. However, immunization coverage with MCV1 has been stagnant with some periods of decline, and uptake of MCV2, introduced in 2013, has been slow. Annual measles incidence remained above the WHO elimination goal of <1/million population. Nationwide measles outbreaks occurred in 2006, 2009, and 2011-2012, reflecting sub-optimal MCV1 vaccine coverage (<95%), an accumulation of susceptible persons in the inter-epidemic years, and delays in preventive, follow-up SIAs. Delays were observed in 2009 and 2012, in particular, when SIAs were conducted later in the year after outbreaks had already occurred. This finding is consistent with findings in Nigeria and Democratic Republic Of Congo, where outbreaks have been observed due to sub-optimal coverage in MCV and SIA [13, 14] and in Zambia, where after a successful SIA a resurgence of cases was observed after 3 years [15]. Experience from the United States indicate that coverage of >90% with two MCV doses are required to end transmission of measles [16].

RI coverage for MCV1 was consistently below the elimination target of 95%. Fluctuations in coverage were recorded with two notable declines noted. These declines can be explained by periods of political instability between December 2007 and March 2008 following post-election violence after the December 2007 general elections. The most recent decline in MCV1 coverage followed the decentralization of health services to the 47 county governments in 2013. During this transition period, there were periods interruption in service delivery due to industrial actions by health workers who were uncertain of how the decentralization would affect them. This demonstrates the need for a functional government and strong health systems for effective vaccine services. Uptake of the second dose of measles vaccine has been low since its introduction into the routine immunization schedule. This may be attributed to failure to follow a systematic process in introducing the vaccine. This led to low awareness among the health workers who provide the service and among care givers who should seek the service. In addition, poor utilization of health services beyond the first year of life and lack of innovative strategies to reach older children may have contributed to low uptake of MCV2. The low uptake in MCV2 was also observed in the initial years after introduction in the United republic of Tanzania [17]. Interventions such as sending out text message reminders to mothers can be used to improve the uptake [18, 19]. In addition, health workers at the immunization service point should inform mothers of the additional dose of the measles vaccine. The non-measles febrile rash surveillance indicator remained > 2 per 100,000 persons nationally indicating that the surveillance system was sensitive. The turnaround time from laboratory was adequate although a decline was observed in 2015. However, there were delays in delivery of blood samples to the laboratory, and this may have been attributed to an inefficient sample shipment system from the county to measles laboratory at Kenya Medical Research Institute. The surveillance program should map out the different transportation mechanisms, including courier services, in different areas of the country to ensure an efficient network of sample shipment. The missing data for some of the variables including age and vaccination status indicates gaps in documentation and this can lead to incorrect estimates. The high number of records missing immunization information could be due to loss of immunization cards, or incomplete history taking by clinicians. Since this was analysis of existing data, some limitations exist. Incomplete datasets and missing data may under-estimate the coverage and number of measles cases reported. Less than half the cases were laboratory confirmed; this might have led to an overestimation of measles incidence, since there are other illnesses that may present like measles, especially rubella. Denominator estimates were based on population projections and might have affected the coverage reported in SIAs and routine immunization, resulting in reported coverage above 100%. The immunization coverage and surveillance indices presented in this paper are national averages and may not show variations at the sub-national levels. The measles surveillance network relies on health facility reported cases and may result in missed cases in the community not seeking health services. However, this analysis was conducted on data from a national population-based surveillance system with laboratory confirmation of cases, and it therefore represents the best assessment of the country's burden of measles disease.

## Conclusion

In conclusion, although there is evidence of progress towards elimination of measles in the country, the relatively stagnant MCV1 coverage and the low uptake of MCV2 could reverse these trends. Efforts to improve MCV1 coverage, strengthen MCV2 uptake, and target high-risk areas (age groups and counties with the higher incidence) need to be put in place. In addition, the Kenya immunization program should work with the county health departments to ensure that challenges in vaccination services are addressed. The indicators show that the surveillance system has performed well over the years; however, there is need to strengthen the system to ensure completeness of information collected and improve the effectiveness of laboratory services. Additionally, there is need to identify counties with weak performance for targeted interventions by the surveillance unit. Incompleteness of information may have led to incorrect estimates and follow up should be made to identify where the gaps are, either at the level of data entry at national level or at the health facility during case notification.

### What is known about this topic

- Measles is a vaccine preventable disease that is targeted for elimination by 2020;
- High vaccination coverage with at least two doses of measles containing vaccine are required to eliminate measles.

### What this study adds

- We found that Kenya has made good progress in implementing measles control and elimination including a robust case based surveillance, conducting supplemental immunization activities and recently the introduction of a second dose of MCV into routine immunization;
- Periods of political instability were followed by a decline in the performance in routine immunization. The country's health system needs to be strengthened and prepared to be able to withstand changes and transitions in governance without negatively affecting service provision.

## Competing interests

The authors declare no competing interests.
